# Exploration of DAPI analogues: Synthesis, antitrypanosomal activity, DNA binding and fluorescence properties

**DOI:** 10.1016/j.ejmech.2017.01.037

**Published:** 2017-03-10

**Authors:** Abdelbasset A. Farahat, Arvind Kumar, Martial Say, Tanja Wenzler, Reto Brun, Ananya Paul, W. David Wilson, David W. Boykin

**Affiliations:** a Department of Chemistry, Georgia State University, Atlanta, GA 30303, United States; b Department of Pharmaceutical Organic Chemistry, Faculty of Pharmacy, Mansoura University, Mansoura 35516, Egypt; c Swiss Tropical and Public Health Institute, Basel 4002, Switzerland; d University of Basel, Basel, 4003, Switzerland

**Keywords:** Diamidines, Stille coupling, Pinner reaction, DAPI, DNA minor groove binders, Antitrypanosomal activity, Fluorescence properties

## Abstract

The DAPI structure has been modified by replacing the phenyl group with substituted phenyl or heteroaryl rings. Twelve amidines were synthesized and their DNA binding, fluorescence properties, *in vitro* and *in vivo* activities were evaluated. These compounds are shown to bind in the DNA minor groove with high affinity, and exhibit superior *in vitro* antitrypanosomal activity to that of DAPI. Six new diamidines (**5b**, **5c**, **5d, 5e, 5f** and **5j**) exhibit superior *in vivo* activity to that of DAPI and four of these compounds provide 100% animal cure at a low dose of 4 × 5 mg/kg i.p. in *T. b. rhodesiense* infected mice. Generally, the fluorescence properties of the new analogues are inferior to that of DAPI with the exception of compound **5i** which shows a moderate increase in efficacy while compound **5k** is comparable to DAPI.

## Highlights

• Twelve amidines were synthesized as analogues to the known antitrypanosomal agent DAPI.
• The DNA binding, fluorescence properties, *in vitro* and *in vivo* activities of the new amidines were evaluated.
• These compounds are shown to bind in the DNA minor groove with higher affinity than DAPI.
• Four new diamidines provided 100% animal cure which is superior to that of DAPI.

## Graphical abstract

**Figure:**
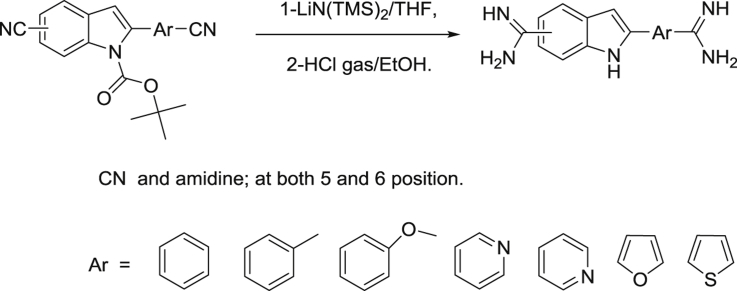

## 1 Introduction

Human African trypanosomiasis also known as sleeping sickness is a neglected tropical disease in Sub-Saharan Africa caused by two subspecies of *Trypanosoma brucei* (*T. b. gambiense* and *T. b. rhodesiense*). It threatens over 65 million people among the poorest in the world in 36 countries [1a]. The trypanosomes are transmitted mainly by blood-feeding tsetse flies. Once infected, the disease generally progresses through two stages which are defined by the location of the parasites in the patient. In the first hemolymphatic stage the parasites reside in the blood and lymph system whereas in the second meningoencephalitic stage the parasites invade additionally the central nervous system [1b]. It is generally accepted that without an effective treatment the disease is lethal. Drugs currently in use for sleeping sickness are pentamidine, suramin, melarsoprol, and a combination of nifurtimox and eflornithine (NECT). The treatment selection is based on the subspecies of *T. brucei* and on the disease stage. All drugs have disadvantages such as difficult administration, serious side effects or resistance issues. Better drugs are needed to control or eliminate sleeping sickness. There are two new molecules, fexinidazole and the oxaborole SCYX-7158 at the stage of clinical development [1c]. However, due to the high attrition rate in drug development it is essential to continue with drug discovery to find new drugs to cure sleeping sickness.

**Fig. 1:**
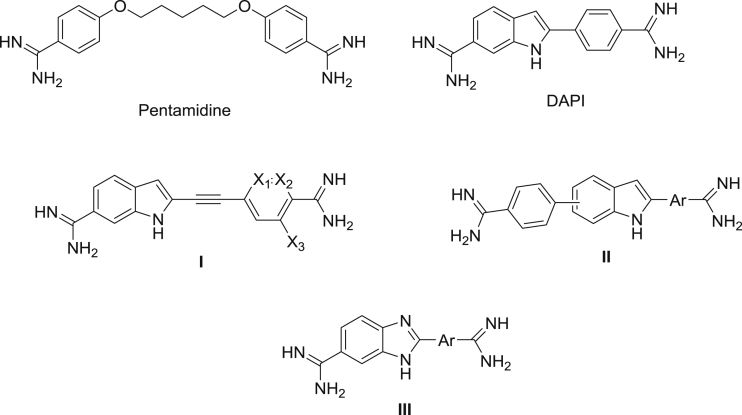
Antitrypanosomal diamidines.

Dicationic amidines that bind in the DNA minor groove are promising agents against African trypanosomiasis. So far, pentamidine (Fig. 1) is the only one of this class which has seen significant human clinical use [2]. However, it is the most frequently used drug for the treatment of first stage sleeping sickness since almost 8 decades and it is still effective by a parenteral route of application.

DAPI (4′, 6-diamidino-2-phenylindole) (Fig. 1), was developed as a compound related to diminazene and stilbamidine, to be used as an antitrypanosomal agent [3]. DAPI has been subsequently found to exhibit a variety of other biological effects, including antifungal, antibacterial, and antiviral activities [3], [4]. Also, DAPI is a fluorescent dye which exhibits several binding modes to DNA [5] and it has been widely utilized as a DNA specific probe for flow cytometry, chromosome staining, DNA visualization and quantitation [6], and has become now an important tool in molecular biology. We previously have reported some phenyl acetylene derivatives of DAPI (compound I, Fig. 1), that showed strong DNA binding and exhibited potent *in vitro* activity superior to that of DAPI [7]. In a related investigation, we extended the overall length of DAPI by adding one phenyl group at the 5 or 6 position of indole and replacing the phenyl group of DAPI with different aryl and heteroaryls (compound II, Fig. 1), these diamidines showed strong DNA binding comparable to that of DAPI and showed stronger *in vitro* antitrypanosomal activity with a comparable or stronger *in vivo* activity than that of DAPI [8]. Moreover, we recently studied the replacement of the indole ring of DAPI with a benzimidazole ring (compound III, Fig. 1), these benzimidazole derivatives showed good *in vitro* activity but exhibited very limited *in vivo* activity [9], which demonstrated the importance of the indole ring of DAPI for the antitrypanosomal activity. Here we report the replacement of the phenyl ring of DAPI with different substituted phenyl and heteroaryl rings in anticipation of finding enhanced *in vivo* activity and physicochemical properties.

## 2 Results and discussion

### 2.1 Chemistry

**Scheme 1:**
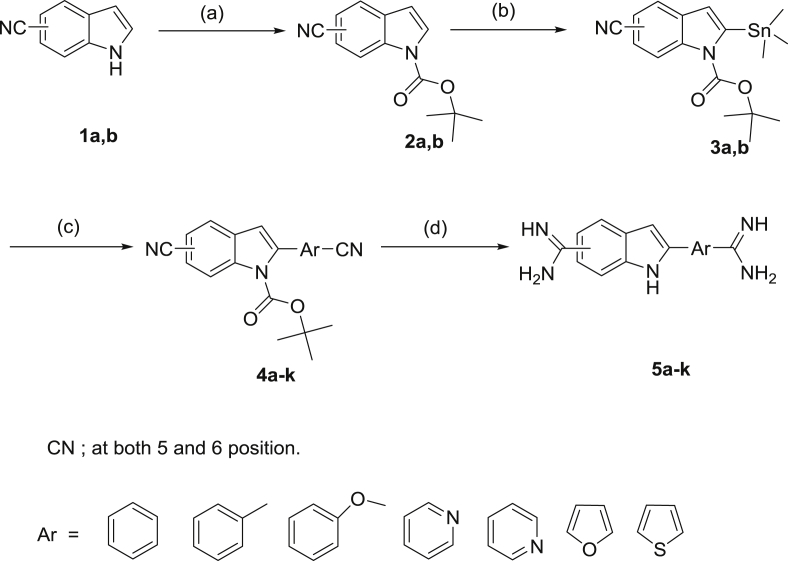
Reagents and conditions. (a) Boc_2_O, DMAP, CH_2_Cl_2_; (b) ClSn(CH_3_)_3_, LDA/THF; (c) Br-Ar-CN, Pd(PPh_3_)_4_, Dioxane (d) 1-LiN(TMS)_2_/THF,2-HCl gas/EtOH.

Scheme 1 describes the preparation of the diamidines **5a-k** in good yield. 5 (6)-Cyanoindoles **1a,b** were protected by employing di-*tert*-butyldicarbonate in dichloromethane using 4-(dimethylamino)pyridine as a catalyst. Lithiation of the Boc-protected indoles **2a,b** was readily achieved with lithium diisopropylamide (LDA) in anhydrous tetrahydrofuran at −20 °C. Subsequent reaction of the lithioindole intermediate with trimethyltin chloride gave the indole stannanes **3a,b** in good yield [10]. The stannane intermediates were allowed to react with different bromoaryl and heteroaryl benzonitriles under Stille coupling conditions in the presence of 5 mol % Pd(PPh_3_)_4_ using dioxane as a solvent to afford the bisnitriles **4a-k** in good yield. The bisnitriles **4a-k** were allowed to react with lithium bis(trimethylsilyl)amide [11] in THF, followed by deprotection of the silylated amidines with ethanolic HCl to furnish the dihydrochloride salts of the diamidines **5a-k**.

**Scheme 2:**
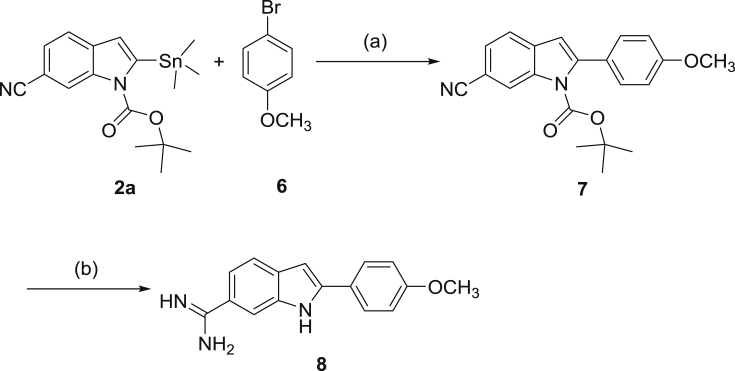
Reagents and conditions: (a) Pd(PPh_3_)_4_, Dioxane (b) i-HCl gas/EtOH.ii-NH_3_/EtOH.

Scheme 2 illustrates the synthesis of the monoamidine **8.** The commercially available 1-bromo-4-methoxybenzene was coupled with the indole stannane **2a** utilizing Stille coupling conditions to afford the mononitrile **7** in high yield. This mononitrile was converted to the monoamidine hydrochloride **8** by utilizing the Pinner methodology [12] by stirring in dry ethanolic HCl to yield the imidate ester hydrochloride which was separated, dried and suspended in ethanolic ammonia to furnish the monoamidine **8**.

### 2.2 Biology

Table 1 summarizes the DNA binding affinities for the twelve new amidino analogues of DAPI as well as both the *in vitro* and *in vivo* activities of these compounds against *Trypanosoma brucei rhodensiense* (*T. b. r.*). For comparative purposes, data for DAPI are included. The thermal melting increase Δ*Tm* (*Tm* of DNA-ligand complex – *Tm* of free DNA) is a rapid and reliable method for ranking binding affinities for large numbers of aryldiamidines [13]. The Δ*Tm* values for the complexes between poly (dA-dT) and the new analogues vary with the change in the substitution of the 2- aryl group and ranges from 10 to >26 °C. The methyl **5b**, methoxy **5d** and the *o*-pyridyl **5e** analogues exhibit high ΔTm values (>26 °C) comparable to that of DAPI. These three compounds have the amidine group at the 6-position of indole, while their isomers with the amidine group at 5-position (**5a**, **5c, 5f**) of indole exhibit lower Δ*Tm* values. We hypothize that this decrease in DNA affinity is likely due to the fact that in the complex 6- amidino indole can form a strong hydrogen bond involving the indole NH to a thymine carbonyl as has been previously reported [7]. While in the analogous complex for the 5-amidino indole such is not possible as the 3-position C—H is pointed into the groove. Removal of the amidine groups from DAPI as in the monoamidine **8** results in a big reduction of binding affinity consistent with the loss of one key cationic binding center.

**Fig. 2:**
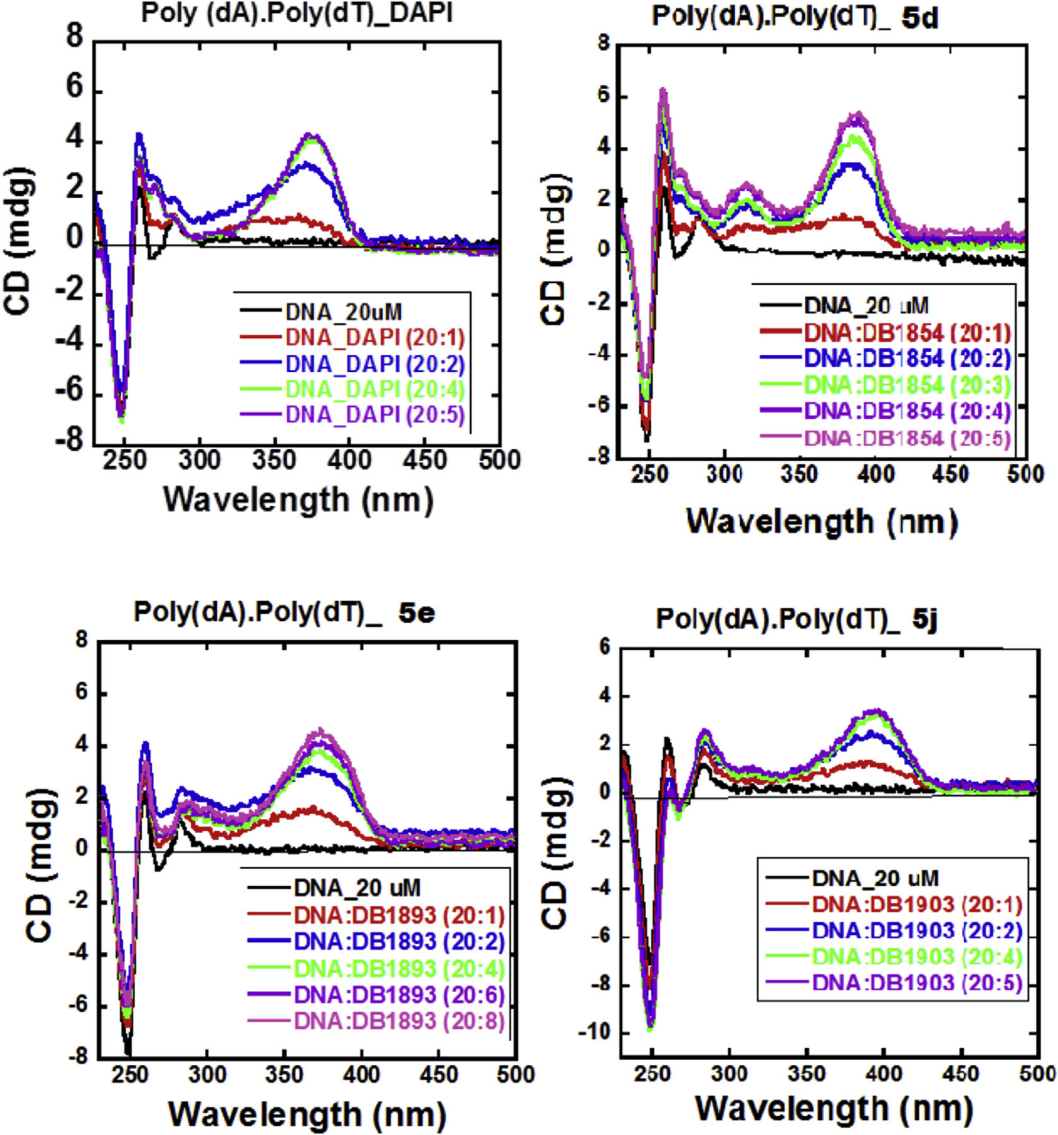
Circular dichroism spectral profiles on the addition of representative compounds, with Poly (dA).Poly (dT) duplex DNA sequences in buffer (10 mM MES, 100 mM NaCl, 1 mM EDTA, at pH, 7.4).

Generally, heterocyclic diamidines have been found to bind in the DNA minor groove in AT base pair sequences of DNA [14].Circular dichroism (CD) spectroscopy is a valuable technique for characterizing minor groove interactions. In order to determine if these new analogues bind in the DNA minor groove, we observed the CD spectroscopy of complex formation. Fig. 2 shows the CD data for the interaction between DNA and **5d**, **5e** and **5j** along with that of DAPI as a reference compound. Minor groove binders yield a large positive induced CD signal on binding to AT sequences and only exhibit small changes in the CD pattern of the DNA spectrum. The induced CD spectrum of DAPI is shown as a reference in Fig. 2(a). The expected positive signal in the compound absorption region with relatively small changes in the DNA region are observed. The results shown in Fig. 2 shows that the CD data are consistent with the Δ *Tm* results for **5d**, **5e** and **5j**. Reflecting strong binding to DNA, compound **5d** exhibits large induced CD spectra in its absorption regions that are higher than that for DAPI while **5e** and **5j** exhibit comparable CD spectra to that of DAPI. This data demonstrates the DNA minor groove binding of these compounds.

DAPI exhibits excellent *in vitro* activity against *T. b. r* (18 nM). All of the newly synthesized compounds, except **5e** and **8,** showed an *in vitro* activity stronger than that of DAPI ranging from a 2–6 fold increase in activity. All the newly synthetized compounds showed good selectivity indices ranging from 133 to 7833 indicating that they are clearly less cytotoxic than DAPI (SI = 48) with exception of compound **8** (SI = 6), which was *in vitro* inactive versus *T. b. r*.

In view of the high *in vitro* activity and selectivity of these new amidines, they were evaluated for efficacy in the acute stage of infection in the rigorous *T. b. r*. STIB900 mouse model (Table 1). The compounds were tested by daily intraperitoneal dosage of 5 mg/kg for four consecutive days. Four of the twelve new compounds (**5d, 5e, 5f** and **5j**) cured all 4 infected mice and gave 100% cure rate. Two compounds (**5b, 5c**) cured 3 out of 4 mice and gave 75% cure rate, while **5g** and **5h** gave 25% cure rate. Compound **5i** was toxic killing 2 out of 4 mice and did not cure any of the two remaining mice. The *in vivo* results for six compounds (**5b**, **5c**, **5d, 5e, 5f** and **5j**) are superior to that of DAPI and pentamidine that is in clinical use for 1st stage HAT since 80 years (both cure 2 out of 4 mice at 4 × 5 mg/kg i.p.) in the STIB900 acute mouse model and therefore the six new compounds merit further evaluation as potential drug candidates for HAT.

The widespread use of DAPI in molecular biology is in large part due to its fluorescence properties. During the study of these new indole amidines we observed that several of the compounds were fluorescent in the visible region. Therefore we decided to record the fluorescence data for the new compounds and compare the results with that of DAPI. These data are presented in Table 2.

The absorption maximum for the new amidines ranges from 312 to 370 nm; that for DAPI is 343 nm. The emission maximum ranges from 418 to 493 nm in comparison with a value of 458 nm for DAPI. A qualitative comparison of quantum yield of emission for the indoles shows that comp **5i** shows a moderate increase in efficacy compared to that of DAPI while compound **5k** is comparable to DAPI. Both new diamidines have the advantage of being less cytotoxic than DAPI (Table 1). Compounds **5b**, **5j** and **8** shows 11–18% decrease from that of DAPI, the rest of the compounds shows significantly lower quantum yield by a factor of 2–25.

## 3 Conclusion

The two diamidines, DAPI and pentamidine are highly active against African trypanosomes. Substitutions on the phenyl ring of DAPI or its replacement with heterocyclic rings results in compounds that bind in the DNA minor groove with high affinity and exhibit superior *in vitro* antitrypanosomal activity to that of DAPI. *In vivo* efficacy of six new diamidines (**5b**, **5c**, **5d, 5e, 5f** and **5j**) was further improved compared to that of DAPI, four of them gave 100% animal cures at the low dose of 4 × 5 mg/kg i.p. Generally, there is a decline in the fluorescence properties of the new analogues compared to that of DAPI with the exception of compound **5i** that shows a moderate increase in efficacy whereas compound **5k** is comparable to DAPI.

## 4 Experimental

### 4.1 Biology

#### 4.1.1 Efficacy studies

The *in vitro* viability assays [15] with the *T. b. r*. strain STIB 900 as well as the efficacy studies in the STIB900 acute mouse model for *T. b. r.* at a 4 × 5 mg/kg i.p. dosage were carried out as previously reported [16].

#### 4.1.2 Tm measurements

Thermal melting experiments were conducted with a Cary 300 spectrophotometer. Cuvettes for the experiment were mounted in a thermal block and the solution temperatures monitored by a thermistor in the reference cuvette. Temperatures were maintained under computer control and increased at 0.5 °C/min. The experiments were conducted in 1 cm path length quartz cuvettes in CAC 10 buffer (cacodylic acid 10 mM, EDTA 1 mM, NaCl 100 mM with NaOH added to give pH = 7.0). The concentrations of DNA were determined by measuring its absorbance at 260 nm. A ratio of 0.3 mol compound per mole of DNA was used for the complex and DNA alone was used as a control [13]. ΔTm values were determined by the peak in first derivative curves (dA/dT).

#### 4.1.3 Circular dichroism (CD)

CD spectra were collected employing a Jasco J-810 spectrometer at different ratios of compound to DNA at 25 °C in MES 10 buffer (10 mM MES, 100 mM NaCl, I mM EDTA). A DNA solution in a 1-cm quartz cuvette was first scanned over the desired wavelength range. DAPI, **5d, 5e** and **5j** at increasing ratios, were titrated into the same cuvette and the complexes rescanned under the same conditions [17].

### 4.2 Chemistry

All commercial reagents were used without purification. Melting points were determined on a Mel-Temp 3.0 melting point apparatus, and are uncorrected. TLC analysis was carried out on silica gel 60 F254 precoated aluminum sheets using UV light for detection. ^1^H and ^13^C NMR spectra were recorded on a Bruker 400 MHz spectrometer (except as noted) using the indicated solvents. Mass spectra were obtained from the Georgia State University Mass Spectrometry Laboratory, Atlanta, GA. If the compounds reported as salts contain a fraction of water and/or solvents, these fractions are seen in HNMR spectra. Elemental analysis were performed by Atlantic Microlab Inc., Norcross, GA. Compounds **5a** [18] and **5d** [19] were previously reported.

## 5 General procedure for the synthesis of the bisnitriles **4a-k** and **7**

Tetrakistriphenylphosphine palladium (0.288 gm, 0.25 mmol) was added to a stirred mixture of the indole stannane (**3a** or **3b**) (5 mmol) and the aryl halide (5 mmol) in de-aireated dioxane (15 mL) under a nitrogen atmosphere. The vigorously stirred mixture was heated at 90–100 °C for 24 h. The solvent was evaporated under reduced pressure, the resulting solid was partitioned between ethyl acetate (200 mL) and 5 mL of concentrated ammonia to remove the palladium complex, washed with water, passed through celite to remove the catalyst, dried over sodium sulfate and evaporated. Purification by column chromatography on silica gel, using hexanes/ethyl acetate (75/25, v/v).

### 5.1 1-(*tert*-Butoxycarbonyl)-2-(2-methyl-4-cyanophenyl)-1*H*-indole-6-carbonitrile (**4b**)

White solid, yield (1.98 gm, 75%). mp 164–164.5 °C; ^1^HNMR (CDCl_3_, 400 MHz) δ 8.66 (s, 1H), 7.67 (d, 1H, *J* = 8 Hz)760–7.58 (m, 2H), 7.55 (br d, 1H, *J* = 8 Hz), 7.41 (d, 1H, *J* = 8 Hz), 6.57 (s, 1H), 2.23 (s, 3H), 1.31 (s, 9H); ^13^CNMR (CDCl_3_, 100 MHz) δ 156.4, 151.1, 149.2, 140.4, 137, 136.1, 132.5, 131.2, 130.0, 129.8, 127.6, 126.2, 125.1, 121.2, 121.1, 119.2, 112.8109.1, 104.2, 27.5, 21.5; ESI-MS: *m*/*z* calculated for C_22_H_19_N_3_O_2_: 357.41, found: 358.2 (M^+^+1); Anal. Calcd. For C_22_H_19_N_3_O_2_: C, 73.93; H, 5.36; N, 11.76. Found: C, 73.69; H, 5.32; N, 11.99.

### 5.2 1-(*tert*-Butoxycarbonyl)-2-(2-methyl-4-cyanophenyl)-1*H*-indole-5-carbonitrile (**4c**)

White solid, yield (0.85 gm, 66%), mp 191–193 °C; ^1^HNMR (DMSO-*d*_6_, 400 MHz) δ 8.11 (d, 1H, *J* = 1.5 Hz), 7.83 (s, 1H), 7.79 (dd, 1H, *J* = 1.5 Hz, *J* = 8.1 Hz), 7.74 (d, 1H, *J* = 8.1 Hz), 7.58 (d, 1H, *J* = 8.4 Hz), 7.47 (dd, 1H, *J* = 1.5 Hz, *J* = 8.4 Hz), 6.88 (d, 1H, *J* = 1.5 Hz), 2.52 (s, 3H), 1.29 (s, 9H); ^13^CNMR (CDCl_3_, 100 MHz) δ 138.2, 137.7, 137.1, 135.9, 134.2, 129.6, 129.4, 127.7, 125.8, 124.5, 120.2, 118.4, 112.4, 110.4, 103.9, 101.5, 27.1, 21.3; ESI-MS: *m*/*z* calculated for C_17_H_11_N_3_: 257 (M^+^).

### 5.3 1-(*tert*-Butoxycarbonyl)-2-(5-cyanopyridin-2-yl)-1*H*-indole-6-carbonitrile (**4e**)

White solid, yield (2.21 gm, 87%). mp 171–171.5 °C; ^1^HNMR (CDCl_3_, 400 MHz) δ 8.96 (d, 1H, *J* = 1.2 Hz), 8.51 (s, 1H), 8.08 (dd, 1H, *J* = 2.0 Hz, *J* = 8.0 Hz), 7.71 (d, 1H, *J* = 8 Hz), 7.69 (d, 1H, *J* = 8 Hz), 7.53 (dd, 1H, *J* = 1.2 Hz, *J* = 8 Hz), 6.95 (s, 1H), 1.44 (s, 9H); ^13^CNMR (CDCl_3_, 400 MHz) δ 155.2, 151.7, 148.8, 140.6, 139.4, 136.9, 131.6, 126.2, 123.1, 122.2, 119.8, 116.4, 112.3, 108.6, 109, 85.6, 27.6; ESI-MS: *m*/*z* calculated for C_20_H_16_N_4_O_2_: 344.37, found: 345.2 (M^+^+1); Anal. Calcd. For C_20_H_16_N_4_O_2_: C, 69.76; H, 4.68; N, 16.27. Found: C, 69.49; H, 4.58; N, 16.08.

### 5.4 1-(*tert*-Butoxycarbonyl)-2-(5-cyanopyridin-2-yl)-1*H*-indole-5-carbonitrile (**4f**)

White solid, yield (2.06 gm, 81%). mp 180–181 °C; ^1^HNMR (CDCl_3_, 400 MHz) δ 8.65 (s, 1H), 7.67 (s, 1H, *J* = 8.0 Hz), 7.61–7.58 (m, 2H), 7.55 (dd, 1H, *J* = 1.6 Hz, *J* = 8.0 Hz), 7.41 (d, 1H, *J* = 8.0 Hz), 6.57 (s, 1H), 1.31 (s, 9H); ^13^CNMR (CDCl_3_, 100 MHz) δ 150.7, 148.9, 139.4, 137.0, 136.5, 133.3, 128.7, 128.5, 127.6, 125.9, 119.3, 117.0, 116.7112.0, 107.2, 86.2, 27.7; ESI-MS: *m*/*z* calculated for C_20_H_16_N_4_O_2_: 344.37, found: 345.2 (M^+^+1); Anal. Calcd. For C_20_H_16_N_4_O_2_: C, 69.76; H, 4.68; N, 16.27. Found: C, 69.69; H, 4.81; N, 15.99.

### 5.5 1-(*tert*-Butoxycarbonyl)-2-(6-cyanopyridin-3-yl)-1*H*-indole-6-carbonitrile (**4g**)

White solid, yield (2.14 gm, 84%). mp 168–168.5 °C; ^1^HNMR (CDCl_3_, 400 MHz) δ 8.81 (s, 1H), 8.35 (d, 1H, *J* = 8.8 Hz), 7.97 (s, 1H), 7.92 (br d, 1H, *J* = 8.0 Hz), 7.81 (d, 1H, *J* = 8.0 Hz), 7.66 (d,1H, *J* = 8.8 Hz), 6.79 (s, 1H), 1.45 (s, 9H); ^13^CNMR (CDCl_3_, 100 MHz) δ 155.0, 151.8, 148.9, 140.7, 139.5, 137.0, 131.2, 126.7, 122.9, 122.4, 119.3, 116.5, 112.4, 108.7, 108.7, 108.4, 85.6, 27.7; ESI-MS: *m*/*z* calculated for C_20_H_16_N_4_O_2_: 344.37, found: 345.2 (M^+^+1); Anal. Calcd. For C_20_H_16_N_4_O_2_: C, 69.76; H, 4.68; N, 16.27. Found: C, 69.66; H, 4.62; N, 16.21.

### 5.6 1-(*tert*-Butoxycarbonyl)-2-(6-cyanopyridin-3-yl)-1*H*-indole-5-carbonitrile (**4h**)

White solid, yield (2.11 gm, 83%). mp 197–197.5 °C; ^1^HNMR (CDCl_3_, 400 MHz) δ 8.81 (s, 1H), 8.34 (d, 1H, *J* = 8.8 Hz), 7.97 (s, 1H), 7.93 (br d, 1H, *J* = 8.0 Hz), 7.81 (d, 1H, *J* = 8.8 Hz), 7.66 (d, 1H, *J* = 8.0 Hz), 6.79 (s, 1H), 1.44 (s, 9H); ^13^CNMR (CDCl_3_, 100 MHz) δ 150.8, 149.1, 139.7, 137.4, 136.3, 132.9, 128.9, 128.3, 127.5, 126.3, 119.2, 116.9, 116.8, 112.0, 107.2, 86.3, 27.7; ESI-MS: *m*/*z* calculated for C_20_H_16_N_4_O_2_: 344.37, found: 345.2 (M^+^+1); Anal. Calcd. For C_20_H_16_N_4_O_2_: C, 69.76; H, 4.68; N, 16.27. Found: C, 69.72; H, 4.66; N, 16.17.

### 5.7 1-(*tert*-Butoxycarbonyl)-2-(5-cyanofuran-2yl)-1*H*-indole-6-carbonitrile (**4i**)

Yellow solid, yield (1.58 gm, 64%). mp 154–155 °C; ^1^HNMR (CDCl_3_, 400 MHz) δ 8.3–8.15 (m, 2H), 7.81–7.74 (m, 2H), 7.20 (s, 1H), 7.14 (br s, 1H), 1.45 (s, 9H); ^13^CNMR (CDCl_3_, 100 MHz) δ 150.7, 148.8, 148.6, 139.0, 129.3, 129.2, 128.3, 127.4, 125.4, 119.6, 116.3, 113.3, 112.7, 112.0, 106.4, 85.9, 27.5; ESI-MS: *m*/*z* calculated for C_19_H_15_N_3_O_3_: 333.34, found: 334.2 (M^+^+1); Anal. Calcd. For C_19_H_15_N_3_O_3_: C, 68.46; H, 4.54; N, 12.61. Found: C, 68.32; H, 4.61; N, 12.53.

### 5.8 1-(*tert*-Butoxycarbonyl)-2-(5-cyanofuran-2yl)-1*H*-indole-5-carbonitrile (**4j**)

Yellow solid, yield (1.7 gm, 69%). mp 162–162.5 °C; ^1^HNMR (CDCl_3_, 400 MHz) δ 8.32 (d, 1H, *J* = 8.4 Hz), 7.94 (s, 1H), 7.66–7.62 (m, 2H), 7.18 (d, 1H, *J* = 3.6 Hz), 6.84 (s, 1H), 1.5 (s, 9H); ^13^CNMR (CDCl_3_, 100 MHz) δ 148.8, 141.5, 139.2, 136.9, 132.0, 128.5, 128.4, 128.3, 125.8, 119.3, 116.6, 113.7, 110.5, 112.9, 107.0, 86.0, 27.7; ESI-MS: *m*/*z* calculated for C_19_H_15_N_3_O_3_: 333.34, found: 334.2 (M^+^+1); Anal. Calcd. For C_19_H_15_N_3_O_3_: C, 68.46; H, 4.54; N, 12.61. Found: C, 68.45; H, 4.56; N, 12.6.

### 5.9 1-(*tert*-Butoxycarbonyl)-2-(5-cyanothiophen-2yl)-1*H*-indole-6-carbonitrile (**4k**)

Dark yellow solid, yield (1.83 gm, 71%). mp 181–181.3 °C; ^1^HNMR (CDCl_3_, 400 MHz) δ 8.31 (d, 1H, *J* = 8.8 Hz), 7.95 (s, 1H), 7.64 (d, 1H, *J* = 8.8 Hz), 7.23 (d, 1H, J = 3.6HZ), 6.95 (s, 1H), 6.76 (d, 1H, *J* = 3.6 Hz), 1.55 (s, 9H); ^13^CNMR (CDCl_3_, 100 MHz) δ 158.8, 150.1, 148.6, 139.2, 129.3, 128.7, 128.1, 126.1, 123.1, 123.1, 119.2, 116.5, 112.6, 112.4, 111.2, 107.0, 85.8, 27.8; ESI-MS: *m*/*z* calculated for C_19_H_15_N_3_O_2_S: 349.41, found: 349.3 (M^+^+1); Anal. Calcd. For C_19_H_15_N_3_O_2_S: C, 65.31; H, 4.33; N, 12.03. Found: C, 65.28; H, 4.41; N, 11.99.

### 5.10 1-(*tert*-Butoxycarbonyl)-2-(4-methoxyphenyl)-1*H*-indole-6-carbonitrile (**7**)

White solid, yield (1.42 gm, 82%). mp 206–207 °C as reported [20]; ^1^HNMR (CDCl_3_, 400 MHz) δ 8.56 (s, 1H), 7.61 (dd,1H, *J* = 2.0 Hz, *J* = 8.0 Hz), 7.51 (d, 1H, *J* = 8 Hz), 7.36 (d, 2H, J = 8.8HZ), 6.99 (d, 2H, J = 8.8HZ), 6.58 (d, 1H, *J* = 2 Hz), 3.89 (s, 3H), 1.38 (s, 9H); ^13^CNMR (CDCl_3_, 100 MHz) δ 159.9, 149.6, 144, 136.3, 132.6, 130, 126.2, 126.1, 121, 119.9, 113.5, 109.2, 106, 84.7, 55.4, 27.6; ESI-MS: *m*/*z* calculated for C_21_H_20_N_2_O_3_: 348.4, found: 349.3 (M^+^+1); Anal. Calcd. For C_21_H_20_N_2_O_3_: C, 72.40; H, 5.79; N, 8.04. Found: C, 72.35; H, 5.68; N, 8.12.

## 6 General procedure for the synthesis of the diamidines **5a-k**

The dinitriles (**4a-k**) (0.66 mmol) were suspended in freshly distilled THF (5 ml), and treated with lithium trimethylsilylamide 1 M solution in tetrahydrofuran (4 ml, 3.98 mmol), the mixture was stirred for three days at room temperature. The reaction mixture was then cooled to zero ^o^C and HCl saturated ethanol (2 ml) was added. The mixture was stirred for two days, diluted with ether and the resultant solid was collected by filtration. The diamidine was purified by neutralization with 1 *N* sodium hydroxide solution followed by filtration of the resultant solid and washing with water and dried. Finally, the free base was stirred with ethanolic HCl for one week to make sure that the (Boc)_2_O group was completely removed, diluted with ether, and the solid formed was filtered and dried to give the diamidines salt.

### 6.1 2-(4-Amidino-3-methylphenyl)-1*H*-indole-6-amidine (**5b**)

Yellow solid, yield (0.149 gm, 61%), mp 277–279 °C; ^1^HNMR (DMSO-*d*_6_, 400 MHz) δ 12.32 (s, 1H), 9.47 (s,2H), 9.32 (s,2H), 9.20 (s,2H), 8.96 (s,2H), 8.01 (s, 1H), 7.92 (s, 1H), 7.83–7.78 (m, 3H), 7.49 (d, 1H, *J* = 8.4 Hz), 6.93 (s, 1H), 2.59 (s, 3H);; ^13^CNMR (DMSO-*d*_6_, 100 MHz) δ 167.0, 165.6, 140.4, 137.1, 136.2, 132.6, 131.2, 130.0, 127.6, 126.2, 121.2, 121.1, 119.2, 112.8, 104.2, 21.6; ESI-MS: *m*/*z* calculated for C_17_H_17_N_5_: 291.35, found: 292 (amidine base M^+^+1); Anal. Calcd. For C_17_H_17_N_5_+2HCl + H_2_O: C, 53.41; H, 5.53; N, 18.31. Found: C, 53.15; H, 5.67; N, 18.12.

### 6.2 2-(4-Amidino-3-methylphenyl)-1*H*-indole-5-amidine (**5c**)

pale solid, yield (0.130 gm, 53%), mp 285–287 °C dec.; ^1^HNMR (DMSO-*d*_6_, 400 MHz) δ 12.39 (s, 1H), 9.55 (s, 2H), 9.35 (s, 2H), 9.32 (s, 2H), 9.12 (s, 2H), 8.23 (s, 1H), 7.93 (s, 1H), 7.87–7.82 (br s, 2H), 7.64 (br s, 2H), 6.95 (s, 1H), 2.59 (s, 1H); ^13^CNMR (DMSO-*d*_6_, 100 MHz) δ 166.5, 165.1, 139.5138.1, 136.5, 136.4, 130.8, 129.3, 127.7, 126.9, 125.8, 121.7, 121.4, 118.7, 112.0, 104.5, 21.3; ESI-MS: *m*/*z* calculated for C_17_H_17_N_5_: 291.35, found: 292 (amidine free base M^+^+1); Anal. Calcd. For C_17_H_17_N_5_+2HCl+0.25H_2_O: C, 55.37; H, 5.39; N, 18.99. Found: C, 55.46; H, 5.43; N, 18.72.

### 6.3 2-(5-Amidinopyridine-2-yl)-1*H*-indole-6-amidine (**5e**)

Yellow solid, yield (0.171 gm, 71%), mp > 300 °C; ^1^HNMR (DMSO-*d*_6_, 400 MHz) δ 12.65 (s, 1H), 9.70 (s,2H), 9.39 (s,2H), 9.13 (s,1H), 9.08 (s, 2H), 8.38 (br s,2H), 8.01 (s, 1H), 7.85 (d, 1H, *J* = 8.4 Hz), 7.51 (s, 1H), 7.46 (d, 1H, *J* = 8.4 Hz); ^13^CNMR (DMSO-*d*_6_, 100 MHz) δ 166.9, 162.0, 146.1, 143.1, 141.3, 136.1, 134.0, 132.3, 127.3, 124.5, 122.1, 118.9, 113.2, 104.3; ESI-MS: *m*/*z* calculated for C_15_H_14_N_6_: 278.31, found: 279.2 (amidine base M^+^+1); Anal. Calcd. For C_15_H_14_N_6_+2HCl+1.35H_2_O+ 0.2 Et_2_O: C, 48.61; H, 5.34; N, 21.52. Found: C, 48.29; H, 4.95; N, 21.24.

### 6.4 2-(5-Amidinopyridine-2-yl)-1*H*-indole-5-amidine (**5f**)

Yellow solid, yield (0.157 gm, 65%), mp > 300 °C; ^1^HNMR (DMSO-*d*_6_, 400 MHz) δ 13.0 (s, 1H), 9.70 (s, 2H), 9.50 (s, 2H), 9.48(s, H), 9.33 (s, 2H), 9.09 (s, 2H), 8.76 (dd,1H, *J* = 2.0 Hz, *J* = 8.0 Hz), 7.51 (d, 1H, *J* = 8.0 Hz), 8.26 (s, 1H), 7.69 (d, 1H, *J* = 8.8 Hz), 7.67 (d, 1H, *J* = 8.8 Hz), 7.55 (s, 1H); ^13^CNMR (DMSO-*d*_6_, 100 MHz) δ 166.7, 161.9, 146.9, 142.6, 140.9, 136.0, 134.7, 131.9, 128.1, 124.0, 122.6, 119.7, 112.7, 104.0; ESI-MS: *m*/*z* calculated for C_15_H_14_N_6_: 278.31, found: 279.4 (amidine base M^+^+1); Anal. Calcd. For C_15_H_14_N_6_+2HCl+1.65H_2_O+0.45Et_2_O: C, 48.7; H, 5.79; N, 20.28. Found: C, 48.81; H, 5.93; N, 19.99.

### 6.5 2-(6-Amidinopyridine-3-yl)-1*H*-indole-6-amidine (**5g**)

Yellow solid, yield (0.171 gm, 71%), mp > 300 °C; ^1^HNMR (DMSO-*d*_6_, 400 MHz) δ 12.85 (s, 1H), 9.68 (s,2H), 9.48 (br s, 3H), 9.32 (s, 2H), 9.07 (s,2H), 8.74 (d,1H, *J* = 8.4 Hz), 7.49 (d, 1H, *J* = 8.4 Hz), 8.25 (s, 1H), 7.70 (d, 1H, *J* = 8.4 Hz), 7.66 (d, 1H, *J* = 8.4 Hz), 7.56 (s, 1H); ^13^CNMR (DMSO-*d*_6_, 100 MHz) δ 166.4, 162.9, 146.9, 142.7, 140.9, 136.0, 134.4, 131.9, 128.2, 124.0, 122.7, 119.7, 112.7, 104.3; ESI-MS: *m*/*z* calculated for C_15_H_14_N_6_: 278.31, found: 279.2 (amidine base M^+^+1); Anal. Calcd. For C_15_H_14_N_6_+2HCl+0.35H_2_O: C, 50.4; H, 4.7; N, 23.5. Found: C, 50.72; H, 4.65; N, 23.16.

### 6.6 2-(6-Amidinopyridine-3-yl)-1*H*-indole-5-amidine (**5h**)

Yellow solid, yield (0.166 gm, 69%), mp > 300 °C; ^1^HNMR (DMSO-*d*_6_, 400 MHz) δ 13.12 (s, 1H), 9.76 (s,2H), 9.51 (s,2H), 9.48 (s,1H), 9.33 (s, 2H), 9.10 (s,2H), 8.75 (d,1H, *J* = 8.4 Hz), 7.51 (d, 1H, *J* = 8.4 Hz), 8.26 (s, 1H), 7.71 (d, 1H, *J* = 8.8 Hz), 7.6 (d, 1H, *J* = 8.8 Hz), 7.56 (s, 1H); ^13^CNMR (DMSO-*d*_6_, 100 MHz) δ 166.9, 162.1, 147.0, 142.7, 141.0, 136.0, 134.2, 132.0, 127.7, 124.1, 122.7, 119.9, 112.8, 104.3; ESI-MS: *m*/*z* calculated for C_15_H_14_N_6_: 278.31, found: 279.2 (amidine base M^+^+1); Anal. Calcd. For C_15_H_14_N_6_+2HCl+0.3H_2_O+ 0.2 Et_2_O: C, 51.08; H, 5.04; N, 22.62. Found: C, 50.94; H, 4.73; N, 22.94.

### 6.7 2-(5-Amidinofuran-2-yl)-1*H*-indole-6-amidine (**5i**)

Yellow solid, yield (0.173 gm, 49%), mp 277–279 °C; ^1^HNMR (DMSO-*d*_6_, 400 MHz) δ 12.98 (s, 1H), 9.70 (s,2H), 9.39 (s,2H), 9.30 (s,2H), 9.07 (s,2H), 8.25 (s, 1H), 7.99 (d, 1H, *J* = 3.6 Hz), 7.73 (d, 1H, *J* = 8.8 Hz), 7.64(b, 1H, *J* = 8.8 Hz), 7.36 (d, 1H, *J* = 3.6 Hz), 7.32 (s, 1H); ^13^CNMR (DMSO-*d*_6_, 100 MHz) δ 166.7, 153.7, 151.6, 140.3, 140.1, 139.1, 130.0, 127.9, 122.7, 121.4, 119.7, 112.4, 110.0, 102.6,; ESI-MS: *m*/*z* calculated for C_14_H_13_N_5_O: 267.29, found: 268.4 (amidine base M^+^+1); Anal. Calcd. For C_14_H_13_N_5_O +2HCl + H_2_O+0.23Et_2_O: C, 47.75; H, 5.18; N, 18.66. Found: C, 47.62; H, 5.48; N, 18.28.

### 6.8 2-(5-Amidinofuran-2-yl)-1*H*-indole-5-amidine (**5j**)

Yellow solid, yield (0.159 gm, 45%), mp 263–265 °C; ^1^HNMR (DMSO-*d*_6_, 400 MHz) δ 12.85 (s, 1H), 9.54 (s,2H), 9.42 (s,2H), 9.31 (s,2H), 9.17 (s,2H), 8.24 (s, 1H), 8.17 (d, 1H, *J* = 4 Hz), 7.91 (d, 1H, *J* = 4 Hz), 7.64 (br s, 2H), 7.11 (br s, 1H); ^13^CNMR (DMSO-*d*_6_, 100 MHz) δ 166.8, 158.9, 142.2, 140.5, 135.7, 133.3, 128.1, 127.7, 126.1, 122.6, 122.3, 119.8, 112.5, 102.6; ESI-MS: *m*/*z* calculated for C_14_H_13_N_5_O: 267.29, found: 267.2 (amidine base M^+^); Anal. Calcd. For C_14_H_13_N_5_O +2HCl+2H_2_O+0.24Et_2_O: C, 45.6; H, 5.47; N, 17.77. Found: C, 45.49; H, 5.62; N, 17.37.

### 6.9 2-(5-Amidinothiophen-2-yl)-1*H*-indole-6-amidine (**5k**)

Yellow solid, yield (0.14 gm, 58%), mp 281–283 °C; ^1^HNMR (DMSO-*d*_6_, 400 MHz) δ 13.0 (s, 1H), 9.61 (s,2H), 9.30 (s,2H), 9.20 (s,2H), 9.03 (s,2H), 8.25 (s,1H), 8.02 (d, 1H, *J* = 4 Hz), 7.76 (dd, 1H, *J* = 1.2 Hz, *J* = 8.4 Hz), 7.61 (d, 1H, *J* = 8 Hz), 7.36 (d, 1H, *J* = 4 Hz), 7.33 (br s, 1H); ^13^CNMR (DMSO-*d*_6_, 100 MHz) δ 166.8, 153.2, 151.9, 140.4, 140.1, 140.0, 130.1, 127.1, 122.9, 121.4, 120.1, 112.5, 110.3, 102.7; ESI-MS: *m*/*z* calculated for C_14_H_13_N_5_S: 283.35, found: 284.1 (amidine base M^+^+1); Anal. Calcd. For C_14_H_13_N_5_S +2HCl+0.02H_2_O: C, 47.14; H, 4.25; N, 19.63. Found: C, 46.96; H, 4.43; N, 19.39.

### 6.10 2-(4-methoxyphenyl)-1*H*-indole-6-amidine (**8**)

The mononitrile **7** (0.23 g, 0.66 mmol) was dissolved in saturated ethanolic HCl (5 mL) and stirred at room temperature for 1 week, isolated from air and moisture. Dry ether was added, the crystals which formed were filtered, dried under vacuum for 1 h, dissolved in absolute ethanol, ammonia gas was passed into the solution for 30 min while cooling and the resulting solution was stirred for 4 days at room temperature. Dry ether was added, the precipitated crystals (HCl salt) were filtered. The diamidine was purified by neutralization with 1 *N* sodium hydroxide solution followed by filtration of the resultant solid and washing with water and dried. Finally, the free base was stirred with ethanolic HCl (5 mL) for 2 days at room temperature, diluted with ether, and the formed solid was filtered and dried.

White solid, yield (0.11 gm, 54%), mp 281–283 °C; ^1^HNMR (DMSO-*d*_6_, 400 MHz) δ 12.03 (s, 1H), 9.26 (s, 2H), 8.97 (s, 2H), 7.92 (m, 3H), 7.69 (d, 1H, *J* = 8.4 Hz), 7.44 (d, 1H, *J* = 8.4 Hz), 7.08 (d, 2H, *J* = 8.4 Hz), 6.95 (s, 1H); ^13^CNMR (DMSO-*d*_6_, 100 MHz) δ 167, 142.8, 137.2, 136.4, 133.5, 129.7, 127.7, 124.3, 120.3, 120, 119.2, 115, 112.2, 55.8; ESI-MS: *m*/*z* calculated for C_16_H_15_N_3_O: 265.31, found: 266.1 (amidine base M^+^+1); Anal. Calcd. For C_16_H_15_N_3_O +1HCl+1.15H_2_O: C, 59.59; H, 5.71; N, 13.03. Found: C, 59.35; H, 5.78; N, 13.01.

## Figures and Tables

**Table 1:** DNA binding, *in vitro* and *in vivo* antitrypanosomal activity of the DAPI analogues.

Compound	Code	ΔTm^a^ (^°^C)	Cytotox^b^ (μM)	T. b. r^c^ (nM)	SI^d^	*In vivo*^f^(cures)
	DAPI	>27	0.86	18	48	2/4
	**5a**	19.2	1.7	3	566	0/4
	**5b**	>26	15.7	5	3140	3/4
	**5c**	15.3	23.5	3	7833	3/4
	**5d**	>26	29.6	6	4876	4/4
	**5e**	22.1	3.2	24	133	4/4
	**5f**	>26	21.3	4	5325	4/4
	**5g**	15.3	2.0	3	666.7	1/4
	**5h**	12.1	1.8	5	394	1/4
	**5i**	11	7.2	9	800	0/2
	**5j**	21.4	2.7	3	900	4/4
	**5k**	10	4.5	11	409	0/4
	**8**	12.6	29	4869	6	ND^e^

a Increase in thermal melting of poly (dA-dT)_n_.

b Cytotoxicity was evaluated using cultured L6 rat myoblast cells.

c The *T. b. r*. (*Trypanosoma brucei rhodesiense*) strain was STIB900. The values are the average of duplicate determinations.

d Selectivity Index for *T. b. r*. expressed as the ratio: IC_50_ (L6)/IC_50_ (*T.b.r*.).

e Not determined as *in vitro* inactive.

f In vivo efficacy was determined in *T. b. rhodesiense* (STIB900) infected mice at 4 × 5 mg/kg i.p. dose.

**Table 2:** Fluorescence data for the new amidines.

Compound	λex^a^,^b^ (nm)	λem^a^,^b^ (nm)	Em. intensity^c^
DAPI	343	458	821
**5a**	322	481	176
**5b**	351	463	611
**5c**	341	461	33
**5d**	351	493	452
**5e**	348	470	461
**5f**	339	462	260
**5g**	342	480	53
**5h**	358	475	200
**5i**	322	418	875
**5j**	348	460	680
**5k**	370	481	810
**8**	312	433	690

a Wavelengths (λ)are indicated for excitation (ex) and emission (em).

b Measurements were made with 0.001 M solutions in distilled water.

c Emission intensities were measured at excitation slit width of 5 and emission slit width of 20.
