# Supplementary material for: Exploration of DAPI analogues: Synthesis, antitrypanosomal activity, DNA binding and fluorescence properties
Source: Eur J Med Chem. 2017 Mar 10;128:70–8. doi: 10.1016/j.ejmech.2017.01.037 (PMC5341734; doi:10.1016/j.ejmech.2017.01.037)
Supplement: supporting information [file mmc1.docx]

**Exploration of DAPI Analogues: Synthesis, Antitrypanosomal activity, DNA binding and Fluorescence properties.**

Abdelbasset A. Farahat^a,b^ *, Arvind Kumar^a^, Martial Say^a^, Tanja Wenzler^c,d^, Reto Brun^c,d^, Ananya Paul^a^_,_ W. David Wilson^a^ and David W. Boykin^a^.

^a^Department of Chemistry, Georgia State University, Atlanta, GA, 30303.

^b^Department of Pharmaceutical Organic Chemistry, Faculty of Pharmacy, Mansoura University, Mansoura 35516, Egypt.

^c^Swiss Tropical and Public Health Institute, Basel, 4002, Switzerland, ^d^University of Basel, Basel, 4003, Switzerland

* Fax: 0014044135505, E-mail: [afarahat@gsu.edu](mailto:afarahat@gsu.edu)
